# Disability, Frailty and Depression in the community-dwelling older adults with Osteosarcopenia

**DOI:** 10.1186/s12877-021-02022-2

**Published:** 2021-01-19

**Authors:** Ki-Soo Park, Gyeong-Ye Lee, Young-Mi Seo, Sung-Hyo Seo, Jun-Il Yoo

**Affiliations:** 1 Department of Preventive Medicine, Institute of Health Sciences, College of Medicine, Gyeongsang National University, Jinju, Korea; 2 Center for Farmer’s Safety and Health, Gyeongsang National University Hospital, Jinju, Korea; 3 Department of Information & Statistics, College of Natural Science, Gyeongsang National University, Jinju, Korea; 4 Department of Orthopaedic Surgery, Gyeongsang National University Hospital, 90 Chilamdong, Jinju, Republic of Korea

**Keywords:** Disability, Frailty, Depression, Osteosarcopenia

## Abstract

**Background:**

The purpose of this study was to investigate the prevalence of osteosarcopenia in the over 60-year-old community and to evaluate whether osteosarcopenia is associated with disability, frailty and depression.

**Methods:**

This study was performed using the baseline data of Namgaram-2, among the 1010 surveyed subjects, 885 study subjects who were 60 years or older and had all necessary tests performed were selected. The Kaigo-Yobo checklist (frailty), World Health Organization Disability Assessment Schedule (WHODAS) and Geriatric Depression Scale-Short Form-Korean (GDSSF-K) were used. The Asian Working Group for Sarcopenia (AWGS 2019) were applied in this study. Osteopenia was measured using data from dual energy X-ray absorptiometry (DEXA) and osteopenia was diagnosed when the T-score was less than − 1.0.

The study subjects were divided into four groups: the normal group, in which both sarcopenia and osteopenia were undiagnosed, osteopenia only, sarcopenia only and the osteosarcopenia group, which was diagnosed with both sarcopenia and osteopenia.

**Results:**

Of the 885 subjects over 60 years old evaluated, the normal group comprised 34.0%, the only osteopenia group 33.7%, the only sarcopenia group 13.1%, and the osteosarcopenia group 19.2%. WHODAS (17.5, 95% CI: 14.8-20.1), Kaigo-Yobo (3.0, 95% CI: 2.6-3.4), and GDSSF mean score (4.6, 95% CI: 3.9-5.4) were statistically significantly higher in the osteosarcopenia group compared the other groups. Partial eta squared (η_p_^2^) of WHODAS (0.199) and Kaigo-Yobo (0.148) values ​​according to Osteosarcopenia were large, and GDSSF (0.096) was medium

**Conclusions:**

Osteosarcopenia is a relatively common disease group in the older adults community that may cause deterioration of health outcomes. Therefore, when evaluating osteopenia or sarcopenia in the older adults, management of those in both disease groups should occur together.

## Background

As people live longer, the world’s population is projected to age rapidly in most regions [1]. Korea became an aging society in 2018, and by 2026 Korea will become a super-aged society [2]. Older adults are increasing the prevalence of many chronic diseases, including osteoporosis and sarcopenia [3].

Although osteoporosis-based clinical outcomes and treatment recommendation studies have been increasingly popular in recent decades, sarcopenia and related studies have been less frequent [4]. In addition, sarcopenia has recently been classified as M62.84 in the ICD-10-CM since 2016 [5].

Sarcopenia is most often seen in metabolic disorders such as diabetes mellitus, obesity, and other serious diseases, including congestive heart failure, chronic renal failure, and chronic obstructive pulmonary disease (COPD) [6–8]. Sarcopenia is substantially correlated with osteopenia (or osteoporosis) in older populations. Several studies have shown that sarcopenia and osteopenia (osteosarcopenia, OS) share similar risk factors and biological pathways [9]. OS is associated with severe physical impairment, which presents a major threat to the loss of freedom in later life. The combination of these two conditions exacerbates adverse health effects and creates a significant problem for the older adults. This combination has been presented as a “hazardous duet” that adds the predisposition of falling from sarcopenia to bone weakness in those with osteopenia [10].

In recent years, questionnaire research tools (World Health Disability Assessment Schedule (WHODAS), Kaigo-Yobo) have been developed to define disability and frailty as health outcomes and to measure these outcomes easily and effectively [11, 12]. OS patients have a higher risk of fracture from falling than those with osteopenia and sarcopenia alone. Studies have also shown that sarcopenia acts as a major risk factor for frailty [13]. However, a study on the relationships among OS, disability and frailty is lacking.

Therefore, the purpose of this study was to investigate the prevalence of OS in the over 60-year-old community and to evaluate whether OS is associated with disability, frailty and depression.

## Methods

### Participants

This study was performed using the baseline data of Namgaram-2, which was developed to study the relationship between the prevalence of musculoskeletal disorders and activity limitations in the older adults in 6 rural villages [14, 15].

At the time of the study, subjects who were diagnosed with chronic diseases and are currently receiving cancer treatment were excluded from the study. Only those with no cognitive problems were included in the study. The cognitive performance evaluation for participants with no cognitive function disorders was performed via an interview study utilizing the Korean version of the Mini-Mental State Examination for Dementia Screening (MMSE-DS) [16]. All surveys were evaluated by a medical professional.

After providing written informed consent, participants completed a questionnaire to assess cognitive function. Subsequently, fasting blood samples were collected from participants, followed by physical function evaluation. Among the 1,010 surveyed subjects, 885 were 60 years or older and had all necessary tests performed. These 885 population were selected as study subjects.

All investigations were conducted after obtaining participant consent and after being reviewed by the Institutional Review Board of our institution (approval number: GIRB-A16-0012).

### Materials

Data on social demographic variables such as gender, age, marital status, economic level perceived by the person, and health behavior variable such as smoking was acquired. Also, survey results, such as one on whether or not the subject was affected by hypertension and diabetes, were used. In addition, the blood test measured hemoglobin, cholesterol, albumin, uric acid, γ-GTP, and creatinine.

#### Frailty (Kaigo-Yobo checklist in Korean older adults)

The Kaigo-Yobo checklist [17] was developed by researchers at the Tokyo Senior Research Institute, and the reliability and validity in Korean older adults have been confirmed [18]. The checklist consists of 15 questions and is a survey instrument that focuses on social activities and evaluation of daily life. The Kaigo-Yobo checklist is a complex domain phenotype aging assessment tool and comprises questions only. The checklist’s 15 items consist of four products for nutritional status; three items for fall; two items for activities; two items for social relationships; and one item each for general health status, communication, mobility, and leisure activities. Furthermore, the appropriate answer to each query was ‘Yes’ or ‘No,‘ and a score of one point was possible for each query. The final score is in the range of 0–15 points, and the higher the score was indicated the higher the degree of frailty.

#### WHODAS-12

The World Health Organization (WHO) developed the WHO Disability Assessment Schedule (WHODAS) based on the International Classification of Functioning, Disability, and Health (ICF). WHODAS-12 measures the difficulties caused by health conditions by dividing the difficulties into six areas (cognition, mobility, self-care, getting along, life activities, and participation) [19].

All questions were measured with a 5-point Likert item system (1 point, not very difficult to 5 points, very severe). The final score is in the range of 0-100 points, and the higher the score, the higher the degree of disability in everyday life.

#### Depressive symptoms

To understand the symptoms of depression in the older adults, the Geriatric Depression Scale-Short Form-Korean (GDSSF-K) adapted and developed for aged people in Korea was used [20]. The GDSSF-K has the advantage of comprising items that are easy for the older adults to understand relative to other current depression measurement tools. This scale of 15 items is graded from 0 to 15 points; higher values indicate worse depression.

#### Osteosarcopenia

Although several criteria have been proposed to define sarcopenia, the recent criteria of the Asian Working Group for Sarcopenia (AWGS 2019) were applied in this study [21].

Dual energy X-ray absorptiometry (DEXA) was used to measure muscle mass. In addition, the measured total appendicular skeletal muscle mass (ASM), excluding bone and fat, divided by the square of the height (m^2^) was calculated (ASM/Ht^2^) and used as the skeletal muscle mass index (SMI). Sarcopenia was defined as SMI less than 7.0 kg/m^2^ in men and less than 5.4 kg/m^2^ in women. Muscle strength was evaluated by grip strength, and the measurement was conducted using the Smedley-type dynamometer (TKK 5401; Takei Scientific Instruments Co., Tokyo, Japan). Both hands were evaluated, twice each. Grip strength was used for analysis as one of the four measured values. The maximum value was used as a reference level, for men below 28 kg and for women below 18 kg.

Osteopenia was measured using data from dual energy X-ray absorptiometry (DEXA) and osteopenia was diagnosed when the T-score was less than − 1.0 [22].

The study subjects were divided into four groups [23]: the normal group, in which both sarcopenia and osteopenia were undiagnosed, and the osteosarcopenia group, which was subdivided into those diagnosed with both sarcopenia and osteopenia, osteopenia only, and sarcopenia only.

### Statistical analysis

The general characteristics of the participants generated descriptive statistics; the chi-square test was conducted on categorical variables, and the ANOVA test was performed on continuous variables. Post-hoc analysis was performed using the Tukey method. The odds ratios (OR) of osteosarcopenia prevalence according to socioeconomic status were analyzed by logistic regression.

The comparison of WHODAS, Kaigo-Yobo, and GDSSF values ​​among the four groups of study subjects occurred after adjustment for gender, age, marital status, economic level, smoking status, hypertension, diabetes, hemoglobin, uric acid, albumin, r-GTP, creatinine, and cholesterol [24–26]. Adjusted means and their 95 % confidence intervals (CIs) were estimated by general linear model (GLM) and the post-hoc test is based on Turkey method. And Partial eta squared (η_p_^2^) was calculated to determine the effect size, using the 0.0099, 0.0588, and 0.1379 considered as small, medium, and large effect sizes [27]. The SAS Version 9.4 program (SAS Institute Inc., Cary, NC) was used as the analysis tool, and the significance level was set to 0.05.

## Result

### General characteristics

Of the 885 older adults people over 60 years old evaluated, 594 (67.1 %) were women. The average participant age was 70.3 ± 6.2 years, 561 (63.4 %) participants had spouses, and 577 (69.7 %) had a higher than average economic status. The smoking rate was 7.5 %, 447 (50.5 %) were diagnosed with hypertension, and 192 (21.7 %) were diagnosed with diabetes.

The diagnosis of osteopenia and osteoporosis (below T-score – 1.0) occurred in 468 subjects (47.1 %), and sarcopenia was diagnosed in 286 (32.3 %) (Table 1).

**Table 1:** General characteristics (Total number = 885)

Variables		Number	%
Sex	Male	291	32.9 %
	Female	594	67.1 %
Age (years)	mean±SD	70.3±6.2	
Spouse	Yes	561	63.4 %
	No	324	36.6 %
Economic status	Low	268	30.3 %
	Middle	465	52.5 %
	High	152	17.2 %
Smoking	Non-smoker	819	92.5%
	Smoker	66	7.5%
Hypertension	No	438	49.5%
	Yes	447	50.5%
Diabetes mellitus	No	693	78.3%
	Yes	192	21.7%

### Osteosarcopenia according to general characteristics

The normal group comprised 34 % (301/885), the only osteopenia group 33.7 % (298/885), the only sarcopenia group 13.1 % (116/885), and the OS group 19.2 % (116/885) (Table 2).

**Table 2:** Osteosarcopenia prevalence according to general characteristics

Variables		Normal		Only Osteopenia		Only Sarcopenia		Osteo-sarcopenia		*p*- value
		N	(%)	N	(%)	N	(%)	N	(%)	
Total		301	34.0	298	33.7	116	13.1	170	19.2	
Sex	Male	172	57.1	75	25.2	26	22.4	18	10.6	<0.001
	Female	129	42.9	223	74.8	90	77.6	152	89.4	
OR (female/male)		OR=1		OR=4.0 (*p*<0.001)		OR=4.6 (*p*<0.001)		OR=11.3 (*p*<0.001)		
Age (years)	mean±SD	68.2±5.1		68.8±5.9		73.1±5.5		75.0±5.8		<0.001
OR (unit: years)		OR=1		OR=1.0 (*p*=0.192)		OR=1.2 (*p*<0.001)		OR=1.2 (*p*<0.001)		
Spouse	Yes	241	80.1	193	64.8	54	46.6	73	42.9	<0.001
	No	60	19.9	105	35.2	62	53.4	97	57.1	
OR (no/yes)		OR=1		OR=2.2 (*p*<0.001)		OR=4.6 (*p*<0.001)		OR=5.3 (*p*<0.001)		
Economic status	Low	76	25.3	87	29.2	45	38.8	60	35.3	0.002
	Middle	153	50.8	168	56.4	56	48.3	88	51.8	
	High	72	23.9	43	14.4	15	12.9	22	12.9	
OR (middle/low)		OR=1		OR=1.0 (*p*=0.090)		OR=0.6 (*p*=0.858)		OR=0.7 (*p*=0.431)		
OR (high/low)		OR=1		OR=0.5 (*p*=0.004)		OR=0.4 (*p*=0.009)		OR=0.4 (*p*=0.003)		
Smoking	No	272	90.4	286	96.0	114	98.3	166	97.6	<0.001
	Yes	29	9.6	12	4.0	2	1.7	4	2.4	
OR (yes/no)		OR=1		OR=0.9 (*p*=0.588)		OR=0.3 (*p*=0.053)		OR=0.6 (*p*=0.194)		
Hypertension	No	141	46.8	178	59.7	46	39.7	73	42.9	<0.001
	Yes	160	53.2	120	40.3	70	60.3	97	57.1	
OR (yes/no)		OR=1		OR=0.6 (*p*=0.160)		OR=1.3 (*p*=0.187)		OR=1.2 (*p*=0.414)		

The distribution of osteosarcopenia by male and female was significantly different (*p* < 0.001), and compared to the normal group, the ORs of the other 3 groups and sex were significantly associated with 4.0 (*p* < 0.001), 4.6 (*p* < 0.001), 11.3 (*p* < 0.001), respectively. and age was statistically higher in the OS group (75 ± 5.8 years, *P* < 0.001) compared to other groups. There was also a statistical difference by spouse’s presence (*p* < 0.001) and at the economic level, the low group had a significant difference of osetosarcoipenia (22.4 %), while the group with a high economic level (14.5 %) (*p* = 0.002). The distribution of osteosarcopenia was also significantly different depending on whether hypertension and diabetes were present (*p* < 0.001).

### WHODAS, Kaigo-Yobo, GDSSF scores according to the presence of osteosarcopenia after adjusting covariates

WHODAS disability mean scores were normal (8.6, 95 % CI: 6.3–11.0), only osteopenia (9.4, 95 % CI: 7.1–11.8), only sarcopenia (16.4, 95 % CI: 13.5–19.3), and OS (17.5, 95 % CI: 14.8–20.1), the OS group had statistically significantly higher WHODAS scores compared to the other groups (*P* < 0.001), and the effect size (partial eta squared (η_p_^2^)) of the WHODAS mean according to the four groups was 0.199, which was large.

Kaigo-Yobo frailty mean scores were normal (1.6, 95 % CI: 1.2-2.0), only osteopenia (1.9, 95 % CI: 1.5–2.2), only sarcopenia (2.7, 95 % CI: 2.3–3.2), and OS (3.0, 95 % CI: 2.6–3.4), the OS group had statistically significantly higher depressive scores compared to the other groups (*P* < 0.001), and the effect size of the frailty means according to the four groups was 0.148, which was large.

The GDSSF depression mean scores were normal (2.7, 95 % CI: 2.1–3.4), only osteopenia (3.1, 95 % CI: 2.4–3.7), only sarcopenia (4.3, 95 % CI: 3.5–5.1), and OS (4.6, 95 % CI: 3.9–5.4), the OS group had statistically significantly higher depressive scores compared to the other groups (*P* < 0.001), and the effect size of the depressive means according to the four groups was 0.096, which was medium (Table 3).

**Table 3:** WHODAS, Kaigo-Yobo, GDSSF score according to the presence of osteosarcopenia

	Normal			Only Osteopenia			Only Sarcopenia			Osteosarcopenia			Effect size	*p* value
	mean	SE	95 % CI	mean	SE	95 % CI	mean	SE	95 % CI	mean	SE	95 % CI	Partial eta squared	
^a^WHODAS	8.6	1.20	6.3–11.0	9.4	1.20	7.1–11.8	16.4	1.48	13.5–19.3	17.5	1.35	14.8–20.1	0.199	< 0.001
^a^Kaigo-Yobo	1.6	0.18	1.2–2.0	1.9	0.18	1.5–2.2	2.7	0.23	2.3–3.2	3.0	0.21	2.6–3.4	0.148	< 0.001
^a^GDSSF	2.7	0.34	2.1–3.4	3.1	0.34	2.4–3.7	4.3	0.42	3.5–5.1	4.6	0.38	3.9–5.4	0.096	< 0.001

^a^Adjusting factors: Age, Sex, Spouse, Economic status, Smoking, Hypertension, Diabetes mellitus, Osteopenia, Sarcopenia, Hb, Uric acid, Albumin, γ-GTP, Creatine, Cholesterol

*WHODAS* World Health Organization Disability Assessment Schedule, *GDSSF *Geriatric Depression Scale-Short Form

### Osteosarcopenia according to the presence of frailty

**Fig. 1:**
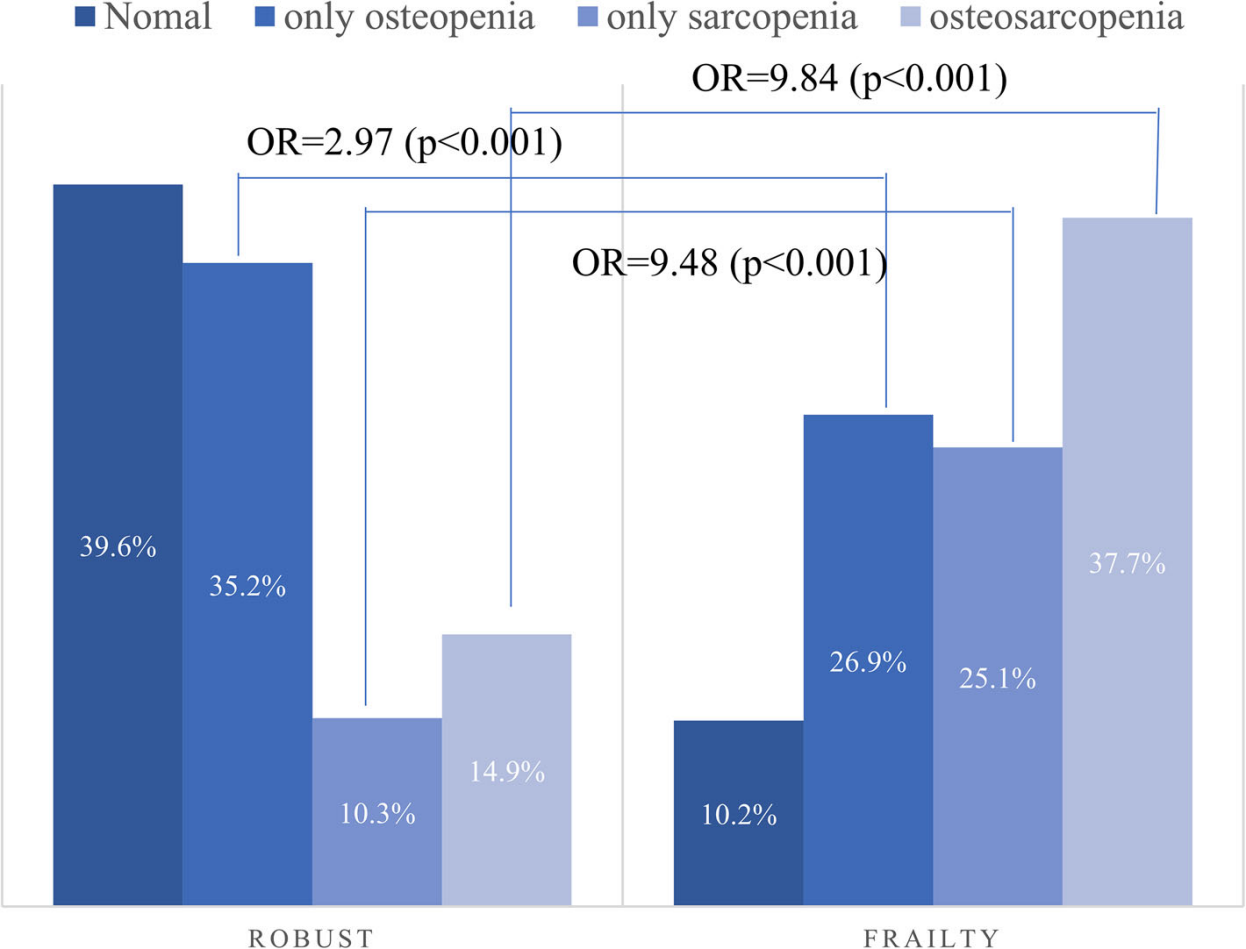
Osteosarcopenia according to the presence of frailty-

The prevalence of OS (37.7 %) in the frailty group was statistically significantly higher than that of OS (14.9 %) in the robust group. (*P* < 0.001) (Fig. 1) And, compared to the robust group, the OR between the normal group and only osteopenia was 2.97 (*p* < 0.001) in the frailty group, the OR with only sarcopenia was 9.48 (*p* < 0.001) and the OR with the osteosarcopenia group was 9.84 (*p* < 0.001).

## Discussion

The main findings of this study were: (1) the prevalence of OS in the community using the new AWGS 2019 criteria was 19.2 %; (2) the prevalence of OS was significantly higher in older women; (3) Hb and albumin levels were lower in subjects with OS; and (4) WHODAS, Kaigo-Yobo, and GDSSF scores were all higher in the OS group after adjusting for general characteristics that were different among the four groups. Therefore, we suggest that osteosarcopenia exacerbates frailty, disability and depression, thereby increasing susceptibility to various chronic diseases.

Huo et al. [23] conducted a cross-sectional study of 680 community-dwelling older individuals and reported that the percentage with OS was almost 40 %. A study of community-dwelling Chinese elders (older than age 65) found the prevalence of OS to be 10.4 % in men and 15.1 % in women. In this study, we observed a prevalence similar to that of community-dwelling Chinese elders. Although the prevalence of OS varies greatly depending on the criteria for diagnosis of osteopenia or sarcopenia, OS is not uncommon in older adults women in the community. In particular, considering the commonality of concomitant osteopenia (including osteoporosis) and sarcopenia in the community, DEXA analyses should measure not only bone density but also muscle mass.

Our results suggest that, in older adults women suspected of being malnourished (low Hb, albumin levels), there may be a risk of impairment due to reduced muscle mass and strength and decreased bone density. There is also a high risk of disability and frailty in these subjects.

WHODAS disability scores were significantly higher in the only osteopenia, only sarcopenia, and OS groups compared to the normal group. Frailty and depression scores were significantly higher in the only sarcopenia and OS groups than in the normal and osteopenia alone groups. Therefore, BMD test performance should be accompanied by the sarcopenia test.

For OS, fall, fracture and mortality rates have been reported to be higher than those with bone loss only or muscle loss only, but other studies have reported different results [28, 29]. However, there are no studies reporting on associations with disability and frailty among these groups. Compared to the osteopenia alone group, the WHODAS score showed a clinically significant difference of 8.8 points when sarcopenia was present suggesting that depression is more likely to impact people with sarcopenia than osteopenia. This is consistent with the results of previous research that identified a correlation between disability, frailty, and depression and sarcopenia. While osteopenia was assessed only by bone density testing, sarcopenia was diagnosed as having a decrease in muscle mass as well as a decrease in muscle strength (function), which may be lower in disability, frailty, and depression. In other words, osteopenia may not have progressed to the point of requiring treatment for assistance in daily life activities. However, sarcopenia may be associated with more activity limitation than osteopenia (not osteoporosis).

This study has several limitations. First, the design was cross-sectional. Therefore, causative relationships could not be ascertained. Second, Because the study subjects are not representative samples, caution should be used when expanding the results of the study.

Nevertheless, our research has strength. The study is meaningful as the first to analyze the relationship between OS and disability, depression, and frailty. Future research should not only focus on OS diagnosis and clinical outcomes such as falls, fractures, and death, but also on the OS patient’s quality of life and the occurrence of disability.

## Conclusions

OS is a relatively common disease in the community and may cause deterioration of health outcomes. Therefore, concurrent evaluation and management of osteopenia and sarcopenia in the older adults is required.

## Data Availability

The datasets used and/or analysed during the current study available from the corresponding author on reasonable request.

## Abbreviations

WHODAS: WHO Disability Assessment Schedule
GDSSF-K: Geriatric Depression Scale-Short Form-Korean
ICD-10-CM: International Classification of Disease, Tenth Revision, Clinical Modification
COPD: Chronic obstructive pulmonary disease
OS: Osteosarcopenia
MMSE-DS: Mini-Mental State Examination for Dementia Screening
ICF: International Classification of Functionin
AWGS: Asian Working Group for Sarcopenia
DEXA: Dual energy X-ray absorptiometry
ASM: Appendicular skeletal muscle mass
SMI: Skeletal muscle mass index
